# COVID-19-Associated Multisystem Inflammatory Syndrome in Children and Cardiovascular Autonomic Control: A Prospective Cohort Study Nine Months after SARS-CoV-2 Infection

**DOI:** 10.3390/jcm13144163

**Published:** 2024-07-16

**Authors:** Paolo Castiglioni, Susanna Rampichini, Carla Giuseppina Corti, Savina Mannarino, Gianvincenzo Zuccotti, Valeria Calcaterra, Damiano Formenti, Andrea Moriondo, Martina Anna Maggioni, Fabio Esposito, Giampiero Merati

**Affiliations:** 1 Department of Biotechnology and Life Sciences (DBSV), University of Insubria, 21100 Varese, Italy; paolo.castiglioni@uninsubria.it (P.C.); damiano.formenti@uninsubria.it (D.F.); giampiero.merati@uninsubria.it (G.M.); 2 IRCCS Fondazione don Carlo Gnocchi, 20148 Milan, Italy; 3 Department of Biomedical Sciences for Health, Università degli Studi di Milano, 20033 Milan, Italy; martina.maggioni@charite.de (M.A.M.); fabio.esposito@unimi.it (F.E.); 4 Department of Cardio-Thoracic-Vascular Diseases, Foundation IRCCS Cà Granda Ospedale Maggiore Policlinico, 20122 Milan, Italy; carlag.corti@gmail.com; 5 Pediatric Department, Buzzi Children’s Hospital, 20154 Milan, Italy; savina.mannarino@asst-fbf-sacco.it (S.M.); gianvincenzo.zuccotti@unimi.it (G.Z.); 6 Department of Biomedical and Clinical Sciences “L. Sacco”, Università degli Studi di Milano, 20157 Milan, Italy; valeria.calcaterra@unipv.it; 7 Department of Internal Medicine and Therapeutics, Università degli Sudi di Pavia, 27100 Pavia, Italy; 8 Department of Medicine and Technological Innovation (DIMIT), University of Insubria, 21100 Varese, Italy; andrea.moriondo@uninsubria.it; 9 Charité—Universitätsmedizin Berlin, Institute of Physiology, Center for Space Medicine and Extreme Environments Berlin, 10117 Berlin, Germany

**Keywords:** MIS-C, autonomic nervous system, heart rate variability, baroreflex sensitivity, pediatric, adolescent

## Abstract

**Background:** Multisystem Inflammatory Syndrome in Children (MIS-C) has emerged as a severe pediatric complication during the SARS-CoV-2 pandemic, with potential long-term cardiovascular repercussions. We hypothesized that heart rate and blood pressure control at rest and during postural maneuvers in MIS-C patients, months after the remission of the inflammatory syndrome, may reveal long-term autonomic dysfunctions. **Methods:** We assessed 17 MIS-C patients (13 males; 11.9 ± 2.6 years, m ± SD) 9 months after acute infection and 18 age- (12.5 ± 2.1 years) and sex- (13 males) matched controls. Heart rate and blood pressure variability, baroreflex function, and hemodynamic parameters were analyzed in supine and standing postures. **Results:** MIS-C patients exhibited reduced heart rate variability, particularly in parasympathetic parameters during standing (pNN50+: 6.1 ± 6.4% in controls, 2.5 ± 3.9% in MIS-C; RMSSD: 34 ± 19 ms in controls, 21 ± 14 ms in MIS-C, *p* < 0.05), with no interaction between case and posture. Blood pressure variability and baroreflex sensitivity did not differ between groups except for the high-frequency power in systolic blood pressure (3.3 ± 1.2 mmHg^2^ in controls, 1.8 ± 1.2 mmHg^2^ in MIS-C, *p* < 0.05). The MIS-C group also showed lower diastolic pressure–time indices (DPTI) and systolic pressure–time indices (SPTI), particularly in standing (DPTI: 36.2 ± 9.4 mmHg·s in controls, 29.4 ± 6.2 mmHg·s in MIS-C; SPTI: 26.5 ± 4.3 mmHg·s in controls, 23.9 ± 2.4 mmHg·s in MIS-C, *p* < 0.05). **Conclusions:** Altered cardiovascular autonomic control may persist in MIS-C patients with, however, compensatory mechanisms that may help maintain cardiovascular homeostasis during light autonomic challenges, such as postural maneuvers. These results highlight the importance of assessing long-term cardiovascular autonomic control in children with MIS-C to possibly identify residual cardiovascular risks and inform targeted interventions and rehabilitation protocols.

## 1. Introduction

The rare Multisystem Inflammatory Syndrome in Children (MIS-C) emerged in early 2020 as a severe pediatric complication during the SARS-CoV-2 pandemic, presenting as an acute inflammatory condition with systemic involvement [1]. The pathophysiology of MIS-C is still unclear [2], but the cardiovascular system is typically affected in the majority of patients, with ventricular systolic dysfunction, troponins elevation, and coronary artery dilation [3]. Hemodynamic instability frequently develops in peripheral vessels, leading in many cases to vasoplegic shock, which requires immediate therapeutic intervention [4]. Although cases have been reported in which symptoms persist or recur, in the majority of patients, the clinical phase usually resolves rapidly [5,6], and the early prognosis after hospitalization and immunomodulation treatment is excellent [7,8]. However, many vascular molecular pathways associated with SARS-CoV-2 infection suggest that the endothelium and microcirculation may play a role even long after MIS-C development [9]. Indeed, in vivo, microvascular damage has been reported throughout the acute and immediate post-acute phases of MIS-C [10]. Therefore, given the parallels between COVID-19 and MIS-C in terms of their inflammatory nature and potential lasting effects, there is growing interest in understanding the post-recovery implications of MIS-C, particularly regarding long-term cardiovascular health.

Unfortunately, while the acute manifestations of MIS-C have been well documented [11], there remains a paucity of data on its long-term consequences on the heart and vessels [12,13,14]. To our knowledge, acute involvement of the cardiac autonomic system, which rapidly reversed with immunoglobulin therapy [15], has been reported anecdotally only in two MIS-C children, and no scientific work has addressed the issue of lasting autonomic cardiovascular control in this disease.

Therefore, we hypothesized that MIS-C may be responsible for a reduced cardiovascular autonomic function that may persist months after the acute infection. Considering the poor information about the pathophysiology of MIS-C and its long-term effects on autonomic function, this study tested our hypothesis by examining the autonomic cardiovascular control at rest and its modulation during a light postural maneuver (lay-to-stand) some months following the onset of symptoms in a cohort of MIS-C children. Such a study may shed light on possible long-term markers of cardiovascular dysfunction in MIS-C patients, to be used for prognostic purposes and as a guide for future interventions and lasting rehabilitation strategies. Heart Rate Variability (HRV) and Blood Pressure Variability (BPV) are valuable physiological methods to evaluate the autonomic control of the cardiovascular system. They provide insights into cardiovascular health and overall well-being, serve as prognostic indicators for cardiovascular events and mortality, and contribute to the risk assessment, disease management, and optimization of treatment strategies across medical and non-medical domains [16].

Therefore, the aim of this study is to assess possible alterations in indices of HRV and BPV, as well as in hemodynamics parameters derived from the blood pressure waveform, nine months after SARS-CoV-2 infection in children who have experienced MIS-C, in comparison to age- and sex-matched controls. By focusing on autonomic control of heart rate and blood pressure and on other hemodynamic parameters during the light autonomic stimulation induced by the postural shift from lying to standing, we aim to elucidate potential mechanisms underlying the long-term cardiovascular sequelae of MIS-C.

## 2. Materials and Methods

### 2.1. Subjects

We enrolled 17 children who had experienced MIS-C following SARS-CoV-2 infection. The inclusion criteria were (a) diagnosis of MIS-C; (b) confirmed prior infection with SARS-CoV-2; (c) age between 6 and 18 years. The exclusion criteria included (a) presence of pre-existing cardiovascular conditions unrelated to MIS-C (e.g., Kawasaki disease); (b) history of other significant systemic illnesses that could compromise the cardiovascular system and the cardiac autonomic regulation; (c) inability to comply with the study procedures; (d) use of medications that could significantly affect the cardiovascular function or autonomic control. We also enrolled 18 controls (CNTR) within the same age range of MIS-C participants, selected among the family members of our institutions’ employees. This inclusion criterion ensures that the control population comes from the same geographical area as the patients (Northern Italy), in particular, that they attended similar schools and meeting places. Exclusion criteria for CNTRs were previous hospitalization after COVID-19 or infection in the two months preceding the study. Given the high spread of SARS-CoV-2 infection in the 6–18 age group in Italy at the time of enrollment, a previous infection was not considered an exclusion criterion. The enrollment period ranged between August and September 2021 for the MIS-C group, and between August and December 2021 for controls. The legal guardians of all minor participants provided informed consent, and the study was approved by the Ethics Committees of the recruiting center (Fondazione don C. Gnocchi, Milan, Italy; Prot. N. 19/21 CE_FdG/FC/SA).

### 2.2. Experimental Procedures

Data were collected in a quiet environment in the morning (from 9 am to 11 am). During the initial interview, the weekly sports activity was habitually carried out before the infection was recorded. Arterial blood pressure was first measured at the brachial artery with an arm cuff in the supine position. Then, continuous noninvasive arterial blood pressure at the finger artery and one lead ECG were recorded with the Finometer^®^ system (Finapres Medical Systems B.V., Amsterdam, Institutenweg 25, 7521 PH Enschede, The Netherlands) for at least 10 min in each of two postures: supine (SUP) and standing (STA). The finger blood pressure signal was calibrated with the Finometer arm cuff following the patented “Return To Flow” technology. The waveform of the arterial blood pressure recorded at the finger site was reconstructed at the brachial site by a digital filter [17] implemented in the Beatscope 1.1a software of Finometer [18]. The participants were asked to empty their bladder before testing and then to stay at rest without speaking during both the SUP and STA recordings. In the postural transition between the SUP and STA positions, particular attention was paid to the following aspects: (1) that the complete transition occurred in a reasonably short time for all patients, i.e., in less than 30 s; (2) that the subject was assisted by an operator in the immediate proximity, in case of fast assistance to episodes of lipothymia or pre-lipothymia; 3) that once the patients had reached the upright position, they were able to maintain the orthostatic conditions on their own for the minutes required by the experimental protocol. Each recording started after an adaptation period to the new posture of at least 5 min. The data analysis focused therefore on the final 5 min of recording in each posture. The R peak was identified on the ECG signal by a threshold-and-derivative algorithm. Parabolic interpolation refined the R wave fiducial point [16]. The R-R interval was calculated beat by beat; premature beats were visually identified and removed to obtain the normal-to-normal sinus node depolarization interval (NNI) series. Movement artifacts and “physiocals” (the Finometer built-in calibrations [19]) possibly present in the blood pressure signal were similarly identified and removed. Systolic blood pressure (SBP) was identified in each beat as the maximum blood pressure value within the corresponding NNI.

- *Heart Rate Variability*. Time- and frequency-domain indices were calculated on the SUP and STA periods separately. Time-domain indices were NNI mean (NNIm) and standard deviation (SDNN); the percentage of NN intervals at least 50 ms longer than their preceding NN interval (pNN50+) [20] or at least 50 ms shorter than their preceding NN interval (pNN50−); and the root-mean-square of successive NN differences (RMSSD) [16]. To derive frequency-domain indices, the NNI series was interpolated evenly at 5 Hz. The power spectrum was estimated by the Welch periodogram with Hann data windows of 180 s length. Hann windows were 90% overlapped and linearly detrended. The periodogram was broadband smoothed by a moving average with order increasing with the spectral frequency [21]. Powers in the very-low-frequency (VLF, between 0.005 and 0.04 Hz), low-frequency (LF, between 0.04 and 0.15 Hz), and high-frequency (HF, between 0.15 and 0.40 Hz) bands were evaluated by integrating the power spectrum. Additionally, the LF/HF powers ratio and the normalized LF power (LFnu, i.e., the LF power divided by the sum of the LF and HF powers) were derived as indices of sympathovagal balance [16]. SDNN is an overall measure of variability, reflecting both the cardiac sympathetic and vagal modulations of heart rate; pNN50+ measures the prevalence of heart rate decelerations due to spikes in vagal outflow, and pNN50- the prevalence of heart rate accelerations due to abrupt vagal withdrawal; RMSSD quantifies the faster components of HRV, which are assumed to mainly reflect vagal heart rate modulations. The HF power is considered a measure of respiratory-driven vagal modulations of heart rate; the LF power reflects the Mayer waves through the baroreflex and is generated by both the sympathetic and vagal cardiac outflows, and the VLF power reflects slow thermoregulatory fluctuations and influences of the renin–angiotensin system [22].
- *ECG-derived respiration*. A respiratory signal was obtained from the fluctuations of the QRS amplitude of the ECG induced by respiratory movements [23]. First, the beat-by-beat series of R peaks was interpolated (5 Hz) and high-pass filtered at 0.02 Hz to remove oscillations too long to be generated by respiratory movements. Then, the power spectrum was calculated, and the breathing rate was estimated as the frequency of the highest spectral peak.
- *Blood pressure variability and baroreflex function*. The SBP power spectrum was similarly calculated on a 5 Hz interpolated series by the Welch periodogram, and the VLF, LF, and HF powers were derived. The SBP-NNI coherency spectrum was estimated from the SBP and NNI spectra and cross-spectrum [21] and averaged over the VLF, LF, and HF bands. The spontaneous baroreflex sensitivity was estimated with the transfer function approach [24] calculating the SBP-RRI transfer function over the LF (BRS_LF_) and HF (BRS_HF_) bands and considering only the spectral lines with a coherency modulus greater than 0.25. The baroreflex function was also assessed with the sequence technique [25]: sequences of >3 consecutive beats in which a blood pressure ramp, i.e., a progressive SBP increase(/reduction), was followed, with a lag of zero, one or two beats, by a progressive lengthening(/shortening) of the pulse interval, were identified, and the slope of the regression line between SBP and pulse interval values in each sequence was taken as a local estimate of baroreflex sensitivity. The local estimates were averaged over SUP and STA to obtain BRS_SEQ_ values. The baroreflex effectiveness index, BEI, was derived as the ratio between the number of sequences and the number of blood pressure ramps.
- *Hemodynamics parameters*. The model-flow method implemented in the Beatscope software of the Finometer was used to derive average hemodynamics parameters in SUP and STA from the beat-by-beat analysis of the blood pressure waveform reconstructed at the brachial site [26]. The Beatscope output parameters belong to three classes: (1) demand/supply indices, which include i. the systolic pressure time index (SPTI) defined as the area under the systolic left ventricular pressure curve, interpreted as the oxygen demand by the myocardium; ii. the diastolic pressure time index (DPTI) defined as the area between the diastolic aortic and left ventricular pressure, interpreted as the oxygen supply to the myocardium; and iii. the DPTI/SPTI ratio, i.e., the ratio between the oxygen demand and supply [27]; (2) cardiac pump indices, namely, the left ventricle ejection time (LVET), stroke volume (SV), and cardiac output (CO); and (3) vascular indices, which are i. the systemic vascular resistances (SVR), ii. the aortic characteristic impedance (Zao), and iii. the arterial compliance (Ca).

### 2.3. Statistics

Preliminary power analysis demonstrated that the sample size is adequate to detect a 20% decrease in the SDNN index (measure of overall HRV over segments of 5 min) at the significance α = 0.05, considering an expected value of 81 (20) ms, as tabulated in [28] for healthy subjects in the age class between 10 and 19 years.

Unpaired *t* statistics described differences between groups in power spectra after log-transformation [29] and coherency spectra after Fisher z-transform [30] to obtain normal distributions. Repeated-measures ANOVA with one independent factor is the general linear statistical model used to compare indices of cardiovascular variability, baroreflex function, and hemodynamics, between cases (MIS-C or CNTR) and postures (SUP or STA). When the factor “case” or the interaction “case” × ”posture” was significant, differences between groups were tested separately in SUP and STA by Fisher’s Least-Significant Difference post hoc analysis. Spectral indices were log-transformed to reduce the skewness of their distribution and coherency indices were z-transformed. When the distributions did not pass the Shapiro–Wilk normality test at *p* > 0.05, the ANOVA on ranks was applied, assessing the significance of the differences between groups in each posture by the Mann–Whitney *U* test. The time-domain indices SDNN, RMSSD, and NNIm, and the hemodynamics indices CO, SVR, Ca, and DPTI had to be log-transformed to pass the normality test. The statistical significance was set at *p* < 0.05. Statistics were performed with the STATISTICA 6.0 Software (StatSoft, Inc., Tulsa, OK, USA).

## 3. Results

### 3.1. General Characteristics

MIS-C participants were studied 287 (80) days after the hospital discharge, as mean (SD). Table 1 reports the demographic characteristics of the participants.

MIS-C and CNTR groups matched well in terms of anthropometric data and gender composition. The two groups were also matched as weekly hours of sports activity performed before the infection: 3.53 (2.96) hours/week in the MIS-C group, 2.97 (2.13) hours/week in the CNTR group, *p* = 0.50.

All the children completed the postural tests. Two out of seventeen (12%) MIS-C children showed slight signs of orthostatic hypotension during the lay-to-stand transition. The symptoms were transient and quickly reversed upon resuming the supine position. Therefore, these two subjects were not excluded from the subsequent analysis. None of such events was detected in controls.

### 3.2. Heart Rate and Blood Pressure Variability

Table 2 compares time-domain indices of Heart Rate Variability between the two groups. The factor “Case” was significant for SDNN, lower in the MIS-C group during SUP and STA, and for pNN50+, pNN50−, and RMSSD, significantly lower in the MIS-C group during STA only.

The two groups had similar respiratory frequencies, which were 0.21 (0.03) Hz in CTRL and 0.22 (0.02) Hz in MIS-C during SUP and 0.20 (0.03) Hz in both groups during STA.

**Figure 1:**
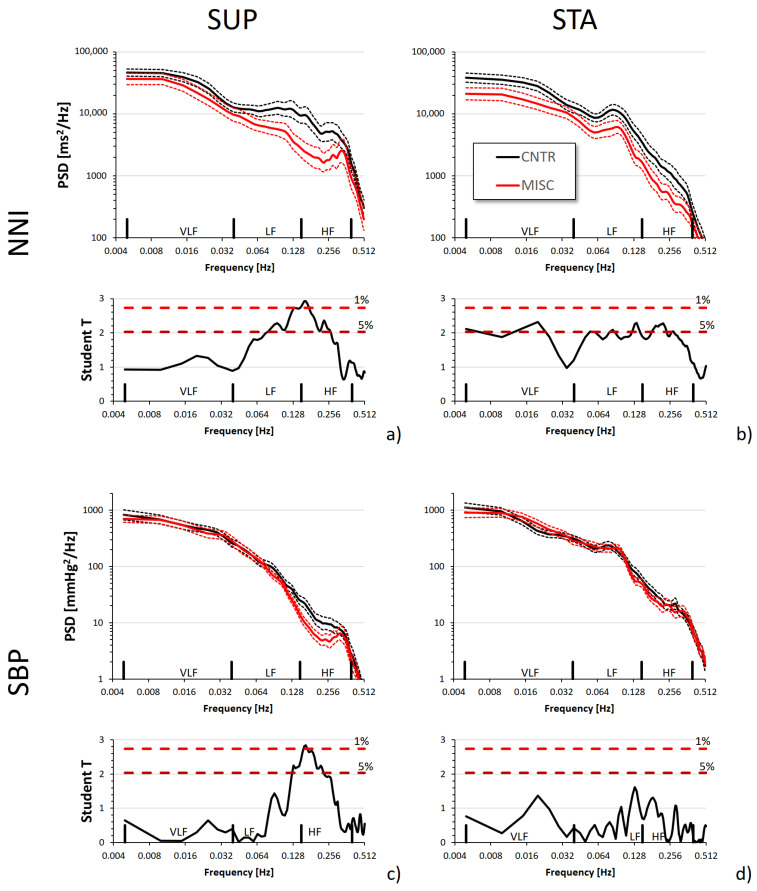
Broadband analysis and Student T statistics of the comparison between MIS-C and CNTR groups for the power spectra of NNI (**a**,**b**) and SBP (**c**,**d**). The upper graph of each panel shows the geometric mean ± geometric SEM in each group; the lower graph shows the T statistics: when T is above the dashed horizontal lines, the difference between groups at the corresponding frequency is significant at *p* < 5% or 1%.

Figure 1 compares power spectra between groups. The NNI power spectrum was depressed in the MIS-C group at around the low- and high-frequency bands in SUP and over almost all the frequencies in STA. By contrast, the SBP spectra differed only in SUP and in the high-frequency band.

Table 3 reports the statistics for indices of spectral analysis. The LF and HF powers of Heart Rate Variability were lower in the MIS-C group in both postures while indices of sympathovagal balance (LF/HF and LFnu) appeared preserved. The HF power of SBP was lower in the MIS-C group during supine rest.

### 3.3. Coherency Spectra and Baroreflex Function

**Figure 2:**
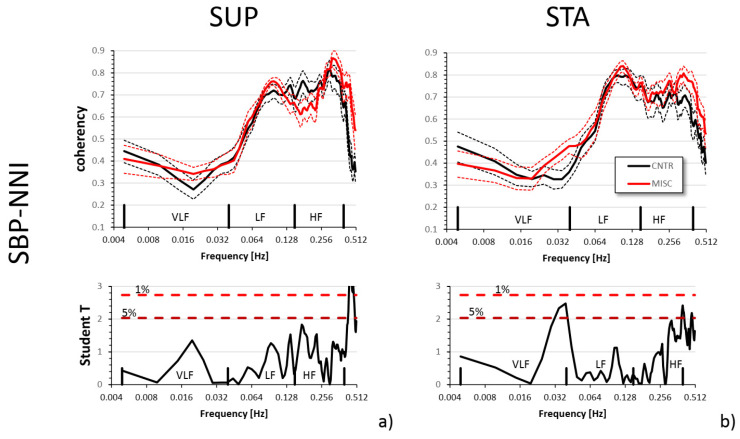
Broadband analysis and Student T statistics of the comparison between MIS-C and CNTR groups for the SBP-NNI coherency in supine (**a**) and standing (**b**) postures. The upper graph of each panel shows the mean ± SEM of the coherency spectra after z-transform in each group; the lower graph shows the T statistics: when T is above the dashed horizontal lines, the difference between groups at the corresponding frequency is significant at *p* < 5% or 1%.

Figure 2 shows the SBP-NNI coherency spectra in MIS-C and CNTR groups separately in the supine and standing postures. The coherency spectra did not show substantial differences between groups.

Differences between groups in the coherency levels or the baroreflex function did not reach statistical significance (Table 4).

Table 5 compares the hemodynamic parameters as obtained by the Model-Flow analysis. The factor case was significant only for indices of myocardial demand or supply: SPTI, significantly lower in the MIS-C group in both postures, and DPTI, significantly lower in the MIS-C group during STA. By contrast, the supply:demand ratio (DPTI/SPTI), cardiac pump indices, and vascular indices did not differ between groups.

## 4. Discussion

This study provides novel insights into the long-term cardiovascular autonomic control in children with MIS-C after several months from the inflammatory process remission, revealing noteworthy differences compared to typically developing children. The first key finding is that even if the heart rate levels are similar in the two groups, both in SUP and STA postures, the overall HRV, as quantified by SDNN, is significantly depressed in children with MIS-C (Table 2). Specifically, indices of the vagal modulations of heart rate calculated in the time domain (pNN50+, pNN50−, and RMSSD, Table 2) and frequency domain (HF power of NNI, Table 3) are lower in the patients in STA posture. These findings are new and suggest a persistent long-term dysregulation of the parasympathetic branch of the autonomic cardiovascular system.

Although such impairment has not been described previously in children, it is well depicted in adult patients who recovered from COVID-19, many of them experiencing some kind of dysautonomia. Long-term observations of HRV in patients with a history of COVID-19 have led to contrasting results, as also parasympathetic overtone and increased HRV have been found [31]. Therefore, it is likely that the variability of cardiac autonomic manifestations in subjects who developed COVID-19 is connected to the time and severity of the acute infection. A review of 17 observational studies highlighted how the progression of dysautonomic damage ranges from a possible acute overtone of the parasympathetic system during the infection up to a long-term period where HRV appears globally depressed, with many patients experiencing symptoms of fatigability and problems of postural adaptation [32]. Another systematic review focused on the long-term consequences of SARS-CoV-2 infection in adults emphasized a parasympathetic inhibition after COVID-19 [33]. As our data in children referred to a post-infection time of about 9 months, they are in agreement with the literature on adults [33], showing a persistently reduced parasympathetic tone in post-infection individuals in the long term. This feature has been connected to a reduction in exercise tolerance [34,35,36] which, in turn, may negatively impact daily physical activities due to an anticipated onset of fatigue.

Conversely, many studies on the post-COVID-19 condition in adults emphasized a parallel increase in sympathetic tone, particularly a greater LF/HF powers’ ratio, index of cardiac sympathovagal balance [37]. Differently from Liviero et al. [37], in our MIS-C children, we did not observe an increased LF/HF powers ratio nor an increased LFnu, another index of cardiac sympathovagal balance. Furthermore, the LF power of SBP, a recognized index of the modulations of the peripheral resistances by the vascular sympathetic outflow [38], which, in our study, reflected nicely the expected postural increase in the vascular sympathetic outflow from supine to standing (Table 3), did not differ at all between groups. Another piece of indirect evidence that the sympathetic tone did not differ between patients and controls is the similar values of LVET, being the ejection time of the ventricle inversely influenced by the myocardial contractility, and thus by the cardiac sympathetic outflow. Finally, no postural tachycardia, a typical sign of cardiac sympathetic overactivity, developed in the MIS-C group following the assumption of the standing position. Therefore, it is unlikely that sympathetic hyperactivity developed over time in our cohort of MIS-C children.

Regarding the possible mechanism of the parasympathetic impairment in MIS-C, there is very little published evidence in the literature to propose solid explanations. However, we can try some speculation and borrow some hypotheses from adult studies, in which the hypothalamic–pituitary–adrenal axis has been demonstrated to be affected by SARS-CoV-2 and could lead to long-term dysautonomia [33]. Moreover, a strong sympathetic activation during the acute disease phases may have produced inflammatory cytokines followed by a powerful anti-inflammatory reflex response to balance this reaction. Such an anti-inflammatory response compensating for the initial hyper-immune reaction is typically regulated by the vagal-cholinergic pathway [39]. The recent observation of a more preserved baroreflex control in COVID-19 individuals during the ICU stay, therefore, in the acute phase of the disease, than in respiratory failure patients without COVID-19 [40], again points toward a more active vagal control, potentially playing an anti-inflammatory role in the context of the hyperinflammatory immune response. As this mechanism usually produces a vagal overtone in the short term, its subsequent suppression may lead to a reduced parasympathetic tone in the long term.

These long-lasting alterations might expose the children to a certain grade of orthostatic or post-exercise hypotension, as in long-COVID adults, in whom an increasing body of evidence suggests a high prevalence of dysautonomia and orthostatic intolerance/tachycardia [41,42,43]. Indeed, two MIS-C children showed slight signs of orthostatic hypotension (consisting of slight dizziness, absence or loss of postural tone and consciousness) during the lay-to-stand transition, while no such events occurred in the control group. However, after assuming the orthostatic posture, the reactivity of the cardiac autonomic nervous system was preserved in individuals with MIS-C, since all HRV indices changed similarly and with the same magnitude in both groups, with no case per condition interaction in HRV parameters (the significance *p* of the interaction between case and posture being always greater than 25%). This suggests that in the long term, the underlying inflammatory condition in individuals with MIS-C did not alter the resilience of the cardiac autonomic nervous system to light autonomic challenges such as postural shifts.

Our quantifications of the baroreflex function, in terms of baroreceptor sensitivity and effectiveness, suggest a substantially preserved baroreflex control in the MIS-C group, with slightly but not significantly lower values of baroreflex sensitivity (Table 4). The change in posture from supine to standing decreased substantially the baroreflex sensitivity, which is an expected postural response to maintaining blood pressure levels while standing. This decrease in baroreflex sensitivity was remarkably similar in the two groups (Table 4). Additionally, the SBP-NNI coherence was similar in patients and controls. Changing posture from supine to standing largely increased the coherence peak at 0.1 Hz in both groups. This 0.1 Hz peak reflects the baroreflex-mediated oscillations in blood pressure induced by modulations of peripheral resistances [44], expected to be more pronounced in the upright posture; also, this finding supports the notion that the autonomic nervous system controls blood pressure similarly in individuals who had MIS-C and in controls during light autonomic stimuli.

By contrast, our study reports, for the first time, a significant and unexpected reduction in the HF power of SBP in the supine posture. Interestingly, the LF and VLF powers did not differ between groups, and during standing, the whole power spectrum of SBP was remarkably similar in patients and controls (Figure 1d). The HF power of SBP is mechanically generated by the respiratory movements of the thorax that modulate the filling of the heart chambers with inspiration and expiration. Sensing such SBP fluctuations, the feedback arm of the baroreflex loop consequently changes the heart rate, inducing a respiratory sinus arrhythmia with spectral components in the HF band. This latter may in turn add its contribution to the blood pressure fluctuations in the HF band through the feedforward arm of the baroreflex, which induces blood pressure changes with changes in the heart rate. Therefore, our results suggest the possibility of an altered cardio-respiratory coupling in our MIS-C group, highlighted during the supine position when the centralization of the blood volume in the thorax might amplify the influence of the breathing movements on the cardiac mechanics. Such autonomic dysregulation may contribute to some hemodynamic instability observed in MIS-C patients, potentially leading to complications such as hypotension.

Hemodynamic parameters derived from the “model flow” approach may provide further insights into the cardiovascular function in MIS-C. Significant differences between patients and controls were found in the pressure–time indices only, with both SPTI and DPTI depressed in the MIS-C group (Table 5). DPTI provides insight into the overall burden on the heart during diastole, which is crucial for coronary blood flow and myocardial perfusion; SPTI reflects the workload on the heart during systole, which is important for cardiac output and tissue perfusion, and depends on the contractile status of the myocardium. A hypothesis to explain the lower SPTI and DPTI values could be possible myocardial damage consequent to the involvement of coronary vessels in the acute phase of the disease. In MIS-C patients, acute cardiac capillary vasoconstrictions may trigger coronary artery aneurysms or lesions, resulting in ischemia followed by ventricular dysfunction and long-term cardiac damage [45]. However, only one of our MIS-C patients had problems related to coronary dilation during the acute phase of the disease, but after a few days of acute therapy, the coronary vessels had a complete restitutio ad integrum. NT-ProBNP and circulating inflammatory indices also had a similar trend in the whole MIS-C group. In addition, all children underwent cardiac MRI in the follow-up control 6 months after acute development, and in no case was there any evidence of myocardial damage or tissue scarring. It is therefore unlikely that a rapidly reversible acute inflammatory condition of the myocardium and a very short-term coronary involvement could have produced a long-term depression of the ability of the left ventricle to develop pressure. Alternatively, we may hypothesize a long-term cardiac deconditioning due to the lack of physical activity consequent to the infection. Actually, cardiopulmonary tests in a 6-month follow-up study demonstrated a deconditioned status in MIS-C children, with shorter exercise duration [46]. It should be noted that even if the level of habitual physical activity performed by MIS-C children before infection was similar to that of controls, as a precaution, they were prohibited from carrying out both leisure and competitive sporting activities in the months following the acute infection. Thus, it is possible that an early readmission to sports and leisure activities would have normalized these indices. This consideration is important in planning loads of physical rehabilitation work that must be designed in the long-term recovery after the acute phase of the infection.

In conclusion, the observed alterations in cardiac autonomic control may have clinical implications. The reduced HRV and autonomic modulation indicate that MIS-C patients may have persistent autonomic dysfunction, which could potentially increase their risk of arrhythmias and other cardiovascular events, especially under stress or during physical activity. In particular, the lower HRV measures during standing posture suggest that MIS-C patients might experience difficulties with orthostatic adaptation, increasing the risk of fainting or dizziness upon standing. As for cardiovascular efficiency, the reduction in myocardial demand indices (SPTI and DPTI) might reflect a compromised ability of the heart to meet increased oxygen demand, potentially leading to exercise intolerance or fatigue in the long term. It seems therefore essential to monitor MIS-C patients over the long term to identify and manage any emerging cardiovascular complications. Regular follow-ups with comprehensive cardiovascular assessments, including HRV analysis and cardio-pulmonary stress testing, might be necessary. Additionally, these findings underscore the importance of a comprehensive cardiovascular assessment in pediatric populations with specific medical conditions to inform personalized management strategies and improve overall health outcomes.

Some limitations of this study should be acknowledged. The sample size was adequate for detecting alterations in the vagal cardiac control and describing accurately the autonomic changes induced by the postural shift; however, a larger number of participants may have clarified aspects now only suggested as trends, as some significances of the case × posture interaction were slightly larger than 0.05. Second, the controls were recruited among the family members of the institution’s employees. This was a practical and feasible approach during that difficult period with the ongoing COVID-19 pandemic, which allowed us to quickly and reliably identify controls who could be readily contacted and willing to participate and could maintain compliance with the study procedures and follow-up. In addition, recruiting from known individuals within the institution ensured adherence to stringent safety protocols while accessing our hospital. However, we cannot completely rule out possible differences between patients and controls related to the families’ educational qualifications. Third, the cross-sectional design precludes causal inferences: longitudinal studies are warranted to elucidate the long-term implications of the lasting altered cardiovascular autonomic control in MIS-C individuals. Further research is needed to explore the underlying mechanisms driving these alterations, for instance, by collecting measurements of peripheral vascular function that could have usefully complemented the blood pressure data, and develop targeted interventions aimed at preserving cardiovascular health in this vulnerable population.

## Figures and Tables

**Table 1:** General characteristics of the participants: mean (SD) with between-groups significance *p*.

	MIS-C	CNTR	*p*
N	17	18	
females/males	4/13	5/13	>0.99
systolic BP (mmHg)	109 (7)	114 (10)	0.15
diastolic BP (mmHg)	69 (7)	72 (8)	0.16
females			
age (years)	11.3 (2.6)	12.2 (1.6)	0.53
height (cm)	154.5 (9.2)	149.1 (14.3)	0.51
body mass (kg)	45.1 (10.5)	45.5 (4.9)	0.95
males			
age (years)	12.5 (2.6)	12.7 (2.6)	0.88
height (cm)	156.8 (12.4)	161.2 (13.7)	0.40
body mass (kg)	51.7 (13.3)	52.5 (14.4)	0.89

*p* by Fisher Exact test for gender composition, by unpaired *t*-test for other variables; systolic and diastolic BP measured with a brachial cuff in the supine position.

**Table 2:** Time-domain Indices of Heart Rate Variability as mean (SD) with factors significance by ANOVA and Fisher LSD post hoc analysis.

	Posture→	SUP	STA	ANOVA *p*-Values		
	Case ↓			Case	Posture	Case × Posture
NNI (ms)	CNTR	844 (90)	655 (93)	0.12	<0.001	0.38
	MIS-C	813 (173)	598 (67)			
		-	-			
SDNN (ms)	CNTR	86 (32)	57 (19)	0.03	<0.001	0.88
	MIS-C	66 (31)	44 (20)			
	post-hoc *p*	<0.05	<0.05			
pNN50+ (%)	CNTR	22.7 (10.9)	6.1 (6.4)	0.04	<0.001	0.96
	MIS-C	16.9 (12.7)	2.5 (3.9)			
	post-hoc *p*	n.s.	<0.05			
pNN50− (%)	CNTR	25.0 (11.6)	5.8 (0.072)	0.03	<0.001	0.88
	MIS-C	18.5 (13.6)	1.8 (0.040)			
	post-hoc *p*	n.s.	<0.05			
RMSSD (ms)	CNTR	86.7 (43.8)	34.0 (19.1)	0.04	<0.001	0.78
	MIS-C	66.1 (47.4)	21.4 (14.2)			
	post-hoc *p*	n.s.	<0.05			

ANOVA on ranks for pNN50+ and pNN50−; post hoc *p* calculated only when the Case factor is significant and reported as n.s. (not significant) when >0.10.

**Table 3:** Spectral indices with factors significance by ANOVA and Fisher’s LSD post hoc analysis.

	Posture→	SUP	STA	ANOVA *p*-Value		
	Case ↓			Case	Posture	Case × Posture
NNI Powers						
VLF (ms^2^)	CNTR	1225 (1.16)	1042 (1.17)	0.09	0.008	0.26
	MIS-C	898 (1.23)	614 (1.24)			
		-	-			
LF (ms^2^)	CNTR	1406 (1.25)	990 (1.2)	0.02	0.016	0.53
	MIS-C	640 (1.28)	518 (1.26)			
	post hoc *p*	<0.05	<0.05			
HF (ms^2^)	CNTR	1695 (1.3)	339 (1.32)	0.02	<0.001	0.62
	MIS-C	608 (1.43)	138 (1.31)			
	post hoc *p*	<0.05	<0.05			
LF/HF	CNTR	0.83 (1.19)	2.92 (1.19)	0.25	<0.001	0.97
	MIS-C	1.05 (1.2)	3.74 (1.15)			
		-	-			
LFnu (%)	CNTR	43 (111)	71 (105)	0.25	<0.001	0.68
	MIS-C	48 (109)	77 (103)			
		-	-			
SBP Powers						
VLF (mmHg^2^)	CNTR	20.9 (1.2)	23.6 (1.2)	0.94	0.10	0.62
	MIS-C	19.6 (1.2)	24.5 (1.2)			
		-	-			
LF (mmHg^2^)	CNTR	10.56 (1.15)	19.4 (1.1)	0.53	<0.001	0.69
	MIS-C	9.24 (1.15)	18.6 (1.1)			
		-	-			
HF (mmHg^2^)	CNTR	3.3 (1.2)	6.7 (1.1)	0.036	<0.001	0.069
	MIS-C	1.8 (1.2)	5.3 (1.2)			
	post hoc *p*	<0.05	n.s.			

Data as geometric mean (geometric SD); post hoc *p* calculated only when the Case factor is significant and reported as n.s. (not significant) when >0.10.

**Table 4:** Indices of SBP-NNI coherency and baroreflex function as Mean (SD) with factors significance by ANOVA.

	Posture→	SUP	STA	ANOVA *p*-Value		
	Case ↓			Case	Posture	Case × Posture
SBP-NNI Coherency						
VLF	CNTR	0.015 (0.01)	0.015 (0.005)	0.46	0.63	0.77
	MIS-C	0.015 (0.01)	0.017 (0.005)			
		-	-			
LF	CNTR	0.087 (0.02)	0.097 (0.02)	0.79	0.01	0.53
	MIS-C	0.088 (0.02)	0.100 (0.02)			
		-	-			
HF	CNTR	0.240 (0.06)	0.208 (0.07)	0.39	0.09	0.17
	MIS-C	0.245 (0.07)	0.241 (0.07)			
		-	-			
Baroreflex Function						
BRS_LF_	CNTR	11.6 (7.0)	5.9 (2.6)	0.11	<0.001	0.18
(ms/mmHg)	MIS-C	8.2 (5.5)	4.9 (2.4)			
		-	-			
BRS_HF_	CNTR	21.4 (12.4)	5.8 (3.3)	0.22	<0.001	0.89
(ms/mmHg)	MIS-C	18.6 (12.41)	4.2 (2.2)			
		-	-			
BRS_SEQ_	CNTR	24.8 (10.6)	9.9 (3.7)	0.08	<0.001	0.49
(ms/mmHg)	MIS-C	20.2 (9.3)	7.4 (2.1)			
		-	-			
BEI (%)	CNTR	72 (11)	57 (17)	0.10	<0.001	0.08
	MIS-C	60 (15)	53 (16)			
		-	-			

Post hoc *p* is calculated only when the Case factor is significant.

**Table 5:** Model Flow parameters: mean (SD) with factors significance by ANOVA and Fisher’s LSD post hoc analysis.

	Posture→	SUP	STA	ANOVA *p*-Value		
	Case ↓			Case	Posture	Case × Posture
Demand/Supply Indices						
SPTI (mmHg·s)	CNTR	31.6 (4.2)	26.5 (4.3)	0.020	<0.0001	0.86
	MIS-C	28.9 (3.1)	23.9 (2.4)			
	post-hoc *p*	<0.05	<0.05			
DPTI (mmHg·s)	CNTR	42.7 (10.4)	36.2 (9.4)	0.048	<0.0001	0.64
	MIS-C	37.2 (12.0)	29.4 (6.2)			
	post-hoc *p*	0.07	<0.05			
DPTI/SPTI	CNTR	1.34 (0.24)	1.33 (0.22)	0.23	0.31	0.35
	MIS-C	1.27 (0.34)	1.20 (0.21)			
		-	-			
Cardiac Pump Indices						
LVET (ms)	CNTR	328 (18)	263 (24)	0.15	<0.0001	0.61
	MIS-C	320 (23)	253 (16)			
		-	-			
SV (ml)	CNTR	45.5 (22.7)	34.0 (16.5)	0.89	<0.0001	0.30
	MIS-C	46.6 (23.5)	31.1 (12.2)			
		-	-			
CO (lpm)	CNTR	3.26 (1.60)	3.20 (1.76)	0.78	0.09	0.57
	MIS-C	3.38 (1.40)	3.11 (1.17)			
		-	-			
*Vascular Indices*						
SVR (MU)	CNTR	1.98 (0.80)	2.25 (0.94)	0.42	<0.0001	0.46
	MIS-C	1.69 (0.64)	1.96 (0.62)			
		-	-			
Z_ao_ (mMU)	CNTR	108.7 (39.4)	107.4 (37.8)	0.71	0.028	0.46
	MIS-C	115.0 (51.7)	112.5 (47.3)			
		-	-			
C_a_ (MU)	CNTR	1.35 (0.62)	1.27 (0.56)	0.77	0.0003	0.63
	MIS-C	1.33 (0.64)	1.20 (0.49)			
		-	-			

SPTI and DPTI = Systolic and Diastolic Pressure Time Index; LVET = Left Ventricle Ejection Time; SV = Stroke Volume; CO = Cardiac Output; SVR = Systemic Vascular Resistance; Z_ao_ = aortic characteristic impedance; C_a_ = arterial compliance; MU = Medical Units; post hoc *p* calculated only when the Case factor is significant.

## Data Availability

The data supporting the main findings of this study are available on the Zenodo repository at https://doi.org/10.5281/zenodo.11122313 (accessed on 15 July 2024) with access granted on justified request to researchers meeting the criteria for access to confidential data.
